# Monitoring heart involvement in treated and untreated transthyretin amyloidosis

**DOI:** 10.1007/s10741-025-10588-x

**Published:** 2025-12-22

**Authors:** Giulio Sinigiani, Paolo Milani, Laura De Michieli, Giuseppe Damiano Sanna, Stefano Perlini, Alberto Cipriani, Giovanni Palladini

**Affiliations:** 1 Department of Cardiac, Thoracic, Vascular Sciences and Public Health, University of Padua, Padova, Italy; 2 Department of Molecular Medicine, University of Pavia, Pavia, Italy; 3 Amyloidosis Research and Treatment Center, Fondazione IRCCS Policlinico San Matteo, Pavia, Italy; 4 Clinical and Interventional Cardiology, Sassari University Hospital, Sassari, Italy; 5 Department of Internal Medicine, University of Pavia, Pavia, Italy; 6 Department of Cardiac, Thoracic, Vascular Sciences and Public Health, University of Padua, Via N. Giustiniani 2, 35121 Padova, Italy

**Keywords:** ATTR, Cardiac amyloidosis, Disease progression, Disease monitoring, Tafamidis

## Abstract

Transthyretin amyloidosis (ATTR) with heart involvement is a progressive, life-threatening condition characterized by amyloid fibril deposition in the myocardium, leading to heart failure, substantial morbidity, and increased mortality. Tafamidis, a transthyretin stabilizer, has been the first and for years the only available disease-modifying therapy, with proven survival benefit. However, a significant proportion of patients continue to experience clinical deterioration, underscoring the need for effective monitoring strategies to guide individual patient management and to serve as surrogate endpoints in clinical trials and in real-world clinical follow up. Current approaches to disease monitoring are largely adapted from heart failure management and include periodic assessment of clinical status, biomarkers, imaging, and functional capacity. Nevertheless, real-world data highlight important limitations, particularly in detecting early disease progression among treated patients. As novel therapies become increasingly available, early identification of suboptimal treatment response is critical to inform timely therapeutic decisions. Developing and validating a reproducible, easy framework for disease monitoring thus remain an urgent research priority. This narrative review summarizes current evidence on monitoring patients with ATTR and heart involvement, including both treated and untreated individuals. It also outlines future directions in this evolving field, emphasizing key knowledge gaps and opportunities for improvement.

## Introduction

Transthyretin amyloidosis (ATTR) is a systemic disease characterized by the deposition of misfolded transthyretin (TTR) in various organs as amyloid fibrils [1]. ATTR occurs in two main forms: hereditary (ATTRv), caused by pathogenic genetic variants, and wild-type (ATTRwt), occurring in the absence of mutations [2]. When amyloid deposition affects the heart, patients show increased left ventricular wall thickness and myocardial stiffness, resulting in heart failure (HF), most commonly with preserved or mildly reduced ejection fraction [3, 4]. Cardiac involvement in ATTR amyloidosis is associated with substantial morbidity and mortality [3].

Tafamidis is a TTR stabilizer that inhibits tetramer dissociation, an early, rate-limiting step in the amyloidogenic cascade, thereby preventing amyloid fibril formation and halting disease progression [5]. In the pivotal ATTR-ACT trial and subsequent long-term extension [6], tafamidis significantly reduced all-cause mortality in patients with cardiac ATTR compared to placebo [7]. As a result, it became the first and, for years, the only disease-modifying therapy approved for clinical use and is now recommended by both the European Society of Cardiology and American College of Cardiology guidelines [8, 9] for ATTR amyloidosis with heart involvement. This adds to the previous indication of tafamidis in treating variant ATTR with neurological involvement.

Real-world data have confirmed its clinical efficacy, leading to stabilization of symptoms, functional status, and quality of life [10–16]. Nonetheless, a considerable proportion of treated patients continue to experience clinical deterioration, and mortality remains substantial. Thus, effective disease monitoring is essential to assess treatment response, detect progression, optimize heart failure management [17, 18], and inform timely therapeutic decisions including a potential transition to emerging TTR-targeted treatments [19, 20], or even combination therapy with agents acting on different and complementary steps of the amyloidogenic cascade (although this approach is not yet supported by clinical data). In addition, validated response and progression criteria could be used as surrogate endpoints in clinical trials.

This narrative review discusses current approaches for monitoring patients with cardiac ATTR, both treated and untreated with disease-modifying drugs, and highlights future directions, emphasizing critical gaps in evidence and areas of unmet need.

## Disease monitoring of untreated ATTR with heart involvement

**Fig. 1:**
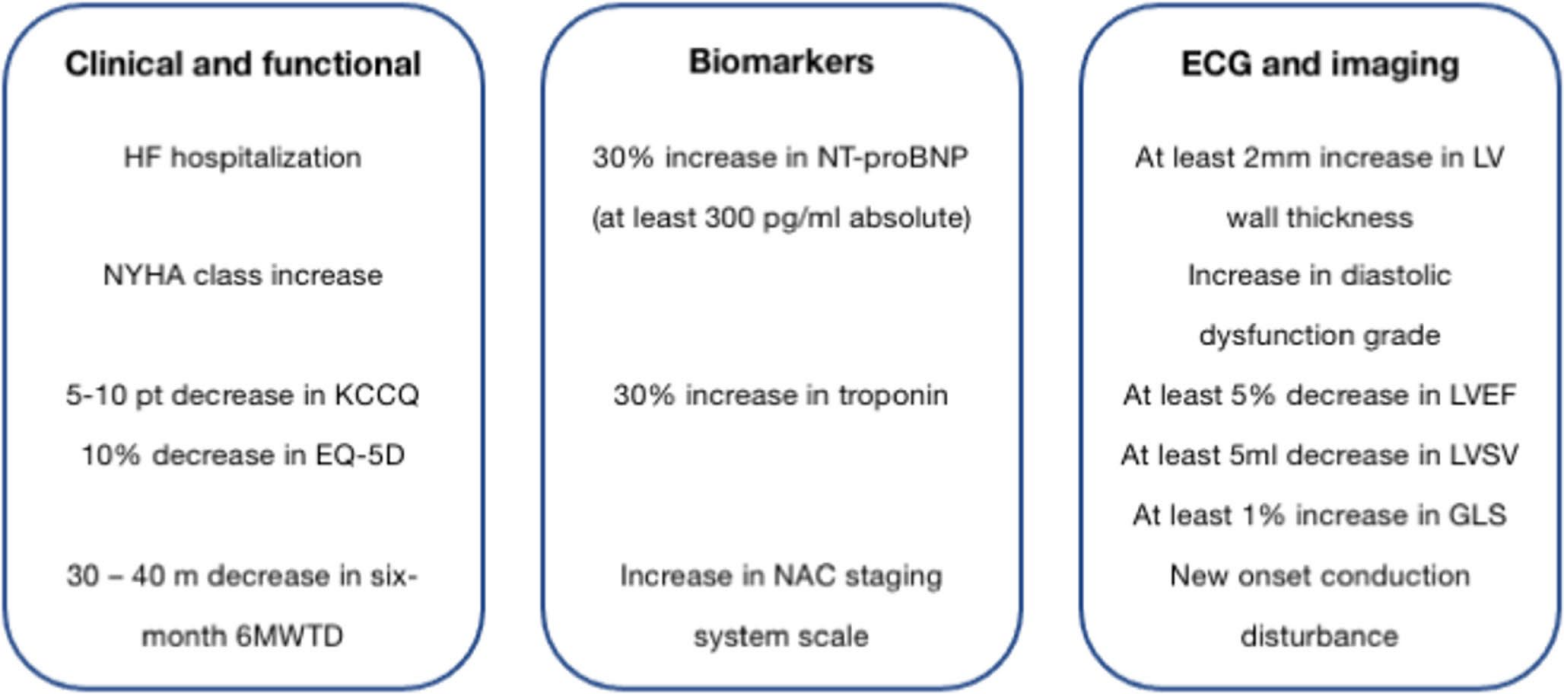
Criteria for disease progression from the European Society of Cardiology. Abbreviations: 6MWTD: six-minute walking test distance; EQ-5D: EuroQol-5D; GLS: global longitudinal strain; HF: heart failure; KCCQ: Kansas City Cardiomyopathy Questionnaire; LV: left ventricle; LVEF: left ventricle ejection fraction; LVSV: left ventricle stroke volume; NAC: National Amyloid Centre; NT-proBNP: N-terminal pro-brain natriuretic peptide; NYHA: New York Heart Association. Modified from Garcia-Pavia et al. [21]

In 2021, the European Society of Cardiology (ESC) proposed a monitoring approach for ATTR with heart involvement that largely mirrors conventional strategies for chronic HF management. The ESC expert consensus identified three core domains for evaluation—clinical/functional status, biomarkers, and imaging—with the goal of achieving a practical yet holistic monitoring strategy (Fig. 1) [21]. It recommended that clinical assessments, biomarker testing, electrocardiographic and imaging evaluations be performed every six to twelve months. Disease progression was defined as the concurrent worsening of at least one parameter from each of the three categories [21]. This model acknowledged the multifactorial nature of ATTR progression, also including the prognostic impact of frailty and non-cardiac comorbidities, although these two relevant factors did not enter the progression/regression algorithm. However, small retrospective studies have shown that this multiparametric approach lacks adequate sensitivity in real-world clinical settings, failing to reliably detect early disease progression in many cases [11, 22, 23]. In addition, most of the proposed variables were selected based on expert consensus and their association with survival outcomes was unproven.

Insights into the natural history of cardiac ATTR have been substantially enriched by longitudinal data from historical cohorts of largely untreated patients. A retrospective analysis of 877 patients followed at the UK National Amyloidosis Centre (NAC) between 2000 and 2020 demonstrated progressive increases in left ventricular (LV) wall thickness, alongside worsening parameters of LV systolic and diastolic function at both 12- and 24-month follow-ups. Interestingly, among these echocardiographic markers – global longitudinal strain (GLS) included - only progressive mitral regurgitation (MR), tricuspid regurgitation (TR) and stroke volume indexed (SVi) were independently associated with all-cause mortality and identified as a robust indicator of disease progression [24]. These results suggest that echocardiography, despite high diagnostic utility, is not able to detect amyloid load changes in ATTR, but only their functional and haemodynamic consequences.

Biomarkers have emerged as a particularly valuable tool in disease monitoring. In a retrospective cohort of 945 patients with ATTR evaluated at NAC between 2011 and 2019, an increase in NAC stage—an integrated score based on NT-proBNP and estimated glomerular filtration rate (eGFR)—was independently associated with increased all-cause mortality at 6-, 12-, and 24-month intervals [25]. These data highlighted the central role of the cardio-renal axis in the natural history of ATTR and provided a rationale for incorporating serial biomarker measurements into clinical practice, as in AL amyloidosis where cardiac biomarkers are key for diagnosis, prognosis, and treatment response [26–28].

Further evidence supporting the prognostic value of biomarker and therapy-response indicators comes from a large multicenter, retrospective study by Ioannou et al., which analyzed 2275 patients with cardiac ATTR, including 321 (14%) receiving tafamidis [29]. At 12 months, an increase in NT-proBNP >700 ng/L and >30%, as well as any initiation or up-titration of loop diuretics—referred to as “outpatient diuretic intensification” (ODI)—were independently predictive of subsequent all-cause mortality. The combination of these two parameters allowed for effective risk stratification and identification of patients at highest risk of poor outcomes. Notably, this study emphasized the potential value of ODI as a pragmatic and objective marker of clinical deterioration that may overcome potential limitations of the New York Heart Association (NYHA) classification, such as interobserver variability, subjectivity, and lack of standardization [30].

Renal function decline has also been shown to correlate with worse outcomes. In a single-center, retrospective study including 2001 ATTR patients—of whom only 78 (4%) were treated with tafamidis—a decline in eGFR ≥ 20% over 12 months was significantly associated with higher all-cause mortality [31]. Importantly, the combined occurrence of NT-proBNP elevation, ODI, and eGFR reduction identified a subgroup of patients at particularly high risk, further reinforcing the relevance of integrated cardio-renal monitoring.

In addition to cardiac and renal markers, functional capacity and frailty are important prognostic indicators [32]. A retrospective study of 2141 patients (97 of whom were treated with tafamidis) evaluated between 2011 and 2023 reported that a >35-meter decline in six-minute walk test distance (6MWTD) over 12 months was significantly associated with increased mortality [33]. When considered alongside NT-proBNP progression and ODI, 6MWTD decline served as a third pillar in identifying patients at highest risk of adverse outcomes. These findings support the inclusion of functional measures in disease surveillance frameworks for cardiac ATTR.

Collectively, these data refine the prognostic significance of parameters long used in the management of chronic HF, allowing their adaptation to the specific trajectory of cardiac ATTR. They also support a multidimensional monitoring strategy—combining imaging, biomarkers, renal and functional parameters—feasible in routine care and reflective of the true complexity of disease progression.

### Disease monitoring of cardiac ATTR on disease-modifying treatment

Current evidence primarily relies on clinical and biochemical markers to assess disease progression during treatment with disease-modifying therapies. A prospective observational study of 339 cardiac ATTR patients from Germany, followed between 2020 and 2023, evaluated the effectiveness of the ESC monitoring criteria. After 12 months of tafamidis therapy, 25 patients (9%) were classified as “non-responders.” However, no significant differences in survival were observed between responders and non-responders [11]. These results should be interpreted with caution, considering the relatively short follow-up and the lack of functional and quality-of-life measures such as 6MWTD and validated questionnaires. Nevertheless, these findings also highlight the inherent complexity of monitoring tafamidis-treated patients, the limited sensitivity of the ESC multiparametric model in real-world clinical practice, and the need for validated monitoring tools that can reliably predict outcomes. The limited sensitivity of the model may arise from several factors. First, the intrinsic multi-domain structure of the model introduces substantial complexity. While this framework is theoretically appropriate for a systemic disorder, in clinical practice a definition of progression based on the alteration of at least one marker across three separate domains may preferentially identify the highest-risk patients and not identify lower-risk patients. Second, several included parameters show considerable intrinsic variability. This is particularly true for echocardiographic indices – as the left ventricular ejection fraction and interventricular septum thickness - but also applies to functional measures – as 6MWTD and Kansas City Cardiomyopathy Questionnaire - which are prone to both technical and physiological fluctuations. Lastly, the imaging markers selected in the score – as left ventricular ejection fraction and diastolic function indices - may not be fully suitable for patients with ATTR cardiomyopathy, as they do not directly quantify myocardial amyloid infiltration, but predominantly reflect haemodynamic consequences. This mismatch may further reduce the ability of the model to discriminate between risk categories in the contemporary context of disease-modifying therapies.

Few studies have explored alternative approaches. In a single-center retrospective study involving 303 cardiac ATTR patients—of whom 271 (90%) were receiving tafamidis—the occurrence of worsening HF, defined as either HF hospitalization or ODI, was assessed over a mean follow-up of 3.3 years. Worsening HF events occurred in 35 patients (11.6%) as hospitalizations and in 145 patients (47.9%) as ODI, both of which were associated with significantly increased all-cause mortality [34]. This study reaffirmed the prognostic relevance of ODI in treated patients and emphasized the importance of HF hospitalization as a marker of disease progression. However, the lack of a standardized evaluation time point limits its utility for prospective, structured monitoring. Furthermore, the use of ODI as a surrogate for worsening heart failure is subject to limitations, primarily due to the heterogeneity in strategies for assessing euvolemia. As a result, diuretic management remains largely unstandardized and often dependent on clinical, subjective, judgment [35]. A second retrospective single-center study, analyzing 238 cardiac ATTR patients treated with tafamidis, applied the definitions of ODI and NT-proBNP progression proposed by Ioannou et al. [29]. Patients showing evidence of either ODI or NT-proBNP elevation at 12 months were at increased risk of all-cause mortality, with the highest risk observed in those who concurrently had both these markers present. The same pattern was noted for the composite endpoint of cardiovascular hospitalizations, defined as acute heart failure admission, use of intravenous diuretics in the emergency department, or unplanned admission for arrhythmia. However, the presence of cardiovascular hospitalizations during the first 12 months was not independently analyzed, limiting conclusions about its temporal relationship with treatment response [36].

In a large multicenter longitudinal study of 683 patients with wild-type ATTR and NYHA class I–II symptoms, previously established progression criteria were applied [16]. Within 12 months, 37 patients (5%) were hospitalized for HF, which was associated with significantly increased subsequent mortality. After excluding patients who were hospitalized or died, 625 individuals (92% of the original cohort) remained evaluable for progression. Among them, approximately one-third exhibited evidence of disease progression. Importantly, only worsening in NYHA functional class (particularly progression to class III), NAC stage, eGFR, NT-proBNP, and ODI were significantly associated with the composite outcome of HF hospitalization and all-cause mortality. In contrast, other commonly used parameters—such as decline of 6MWTD or left ventricular ejection fraction, worsening of interventricular septal thickness or E/e′ ratio—were not predictive in this population. Based on these findings, two predictive models were developed: one based on clinical progression to NYHA class III and NAC stage worsening; and a second model incorporating ODI, eGFR decline, and NT-proBNP elevation. Both models demonstrated comparable accuracy in predicting adverse outcomes. These results collectively suggest that disease monitoring during tafamidis treatment should prioritize clinical and laboratory parameters—specifically those reflecting haemodynamic congestion (ODI, NYHA class, NT-proBNP), neurohormonal activation (NT-proBNP), and renal function (eGFR). Notably, these parameters could also be evaluated individually within a unified model. Such an approach may help overcome the intrinsic limitations of the NAC score — whose historical derivation and validation cohort were untreated — and may improve risk discrimination, particularly for low- and intermediate-risk patients receiving disease-modifying treatments [37]. Although these data are currently limited to patients with wild-type ATTR and mild symptoms (NYHA class I–II), they represent the first effort toward establishing a comprehensive, feasible, and evidence-based framework for monitoring treatment response and disease progression in this setting [38].

By contrast, the role of ECG and imaging in assessing disease progression during treatment with disease-modifying therapies remains uncertain. In studies applying the ESC strategy for progression assessment, only a minority of patients met ECG-based progression criteria. Moreover, no dedicated investigations have systematically evaluated ECG parameters as tools for monitoring progression, representing a major gap in current knowledge. Conversely, more evidence is available for cardiac imaging. Ideally, an imaging parameter should not only quantify amyloid load and its progression, but also capture the resulting structural, functional, and biomarker alterations, and ultimately provide robust prognostic information. In this context, echocardiography has not proven capable of fulfilling this role, as its parameters mainly reflect secondary functional and haemodynamic consequences of amyloid load changes [11, 16, 24].

By contrast, bone scintigraphy has demonstrated reliability in detecting amyloid burden at diagnosis. For longitudinal monitoring, data from clinical trials reported a reduction in cardiac tracer uptake after treatment with vutrisiran [39] and with the NI006 antibody [40], suggesting a potential role in tracking changes in amyloid load. However, a subsequent case report showed that, despite a reduction in cardiac uptake, amyloid burden assessed by positron emission tomography remained unchanged after 13 months of vutrisiran therapy [41], and this finding was not associated with improvements in haemodynamic or laboratory parameters. These limitations were confirmed in a study of 66 treated patients (64% patisiran, 21% inotersen, and 15% tafamidis), in which 21% demonstrated a reduction in cardiac uptake on follow-up scans after a median of 28 months. Notably, among the 28 patients with improved scans, many still exhibited disease progression according to established biochemical criteria [42]. These findings underscore the controversial clinical utility of bone scintigraphy in tracking disease course, as it may fail to detect changes in amyloid load and to capture the accompanying structural, functional, and biomarker alterations.

A potential role for tracking disease progression was suggested for extracellular volume (ECV) assessed on cardiac magnetic resonance (CMR). In light-chain (AL) amyloidosis, both an ECV increase and a reduction of at least 5% at 12 months were associated with clonal response—indirectly reflecting amyloid load [43]—as well as with structural, functional, and biomarker alterations. Moreover, these changes demonstrated prognostic relevance [44]. In ATTR, robust evidence is still lacking, except for a recent study of 189 patients, including 70 treated with patisiran, in which an ECV increase ≥ 5% was associated with adverse prognosis. In addition, among treated patients, structural and functional parameters—but not biomarkers—improved, and 6% demonstrated ECV regression, defined as a ≥ 5% reduction [45]. This coexistence of ECV reduction and morpho-functional improvement was also reported in patients treated with acoramidis [46]. These findings suggest that CMR-derived ECV may represent an optimal imaging parameter for ATTR amyloidosis monitoring, although confirmation in larger studies is warranted.

In summary, available evidence confirms that several markers previously validated in untreated cardiac ATTR retain an important prognostic value in treated patients. However, an integrated, standardized, and widely applicable monitoring strategy remains to be fully defined.

## Future directions

Despite increasing research efforts and the emergence of real-world data (Table 1), a standardized and comprehensive framework for reliably assessing disease progression in cardiac ATTR still remains elusive. This gap largely stems from the multisystemic nature of the disease, in which the pattern, severity, and rate of organ involvement vary significantly among patients. In AL, simple and effective criteria for assessing disease progression and response to therapy are well established, primarily based on serial measurements of NT-proBNP, free light-chains and troponin levels. A similar laboratory-based strategy for disease monitoring in ATTR with cardiac involvement may be envisioned, potentially incorporating serial assessments of TTR levels. This approach is supported by a post-hoc analysis of the ATTRibute-CM trial, in which an early and sustained increase in serum TTR levels by acoramidis independently predicted improved survival [47]. However, important differences between AL and ATTR should be acknowledged. First, ATTR, particularly the wild-type form, predominantly affects older, often multimorbid individuals, leading to distinct clinical and epidemiological profiles. Second, the disease course of ATTR is typically more indolent and slow-progressing compared to AL. Third, while regression of cardiac involvement is well documented with effective AL therapies, the capacity of current treatments for ATTR to reverse disease course remains less clearly established. Moreover, the extent of biomarker increase that is observed in ATTRwt is lower when compared with AL, probably due to a somewhat lower cardiac toxicity of amyloid deposition. Therefore, changes in NTproBNP release might be less evident when either improvement or deterioration take place. In this context, the ideal approach to developing a reliable monitoring model for ATTR should begin with the identification and validation of biochemical markers combined with imaging, whose longitudinal changes can predict adverse outcomes. Based on available evidence, those reflecting haemodynamic congestion, neurohormonal activation, and renal function appear to be the most promising candidates. A pragmatic model should therefore prioritize the inclusion of the most predictive and easily obtainable markers. This could be particularly true for specific patient subgroups, such as the very elderly (over 80 years) or those with significant multimorbidity. Identifying these subpopulations could therefore be of considerable importance for refining individualized monitoring strategies.

**Table 1:** Progression criteria for ATTR amyloidosis with heart involvement

Parameters	Study	ATTR subtype	On treatment	Outcome
MR and TR grade, SVi	Chacko et al. [24]	565 ATTRwt 312ATTRv	-	All-cause mortality
ECV on CMR	Patel et al. [44]	100 ATTRwt 89 ATTRv	70 (39%)	All-cause mortality
NAC score	Law et al. [25]	727 ATTRwt 218 ATTRv	-	All-cause mortality
NT-proBNP or/and ODI	Ioannou et al. [30]	1,718 ATTRwt 557 ATTRv	321 (14%)	All-cause mortality
eGFR	Ioannou et al. [31]	1,385 ATTRwt 616 ATTRv	78 (4%)	All-cause mortality
6MWTD	Ioannou et al. [33]	1,573 ATTRwt 568 ATTRv	97 (5%)	All-cause mortality
ESC criteria	Aus dem Siepen et al. [11]	308 ATTRwt 31 ATTRv	339 (100%)	All-cause mortality and heart transplantation
HF hospitalization or ODI	Zeldin et al. [34]	303 ATTR	271 (90%)	All-cause mortality
NT-proBNP	Bampatsias et al. [36]	201 ATTRwt 37 ATTRv	238 (100%)	All-cause mortality and CVH
HF hospitalization NYHA class, NAC score NT-proBNP, ODI, eGFR	Sinigiani et al. [16]	683 ATTRwt in NYHA class I-II	683 (100%)	All-cause mortality and HF hospitalization

*6MWTD* six-minute walking test distance; *ATTRv* hereditary transthyretin amyloidosis; *ATTRwt* wild-type transthyretin amyloidosis; *CMR* cardiac magnetic resonance; *CVH* cardiovascular hospitalization; *ECV* extracellular volume; *eGFR* estimated glomerular filtration rate; *ESC* European society of cardiology; *HF* heart failure; *MR* mitral regurgitation; *NAC* National Amyloid Centre; *NT-proBNP* N-terminal pro-brain natriuretic peptide; *NYHA* New York Heart Association; *ODI* outpatient diuretic intensification; *Svi* stroke volume indexed; *TR* tricuspid regurgitation

**Fig. 2:**
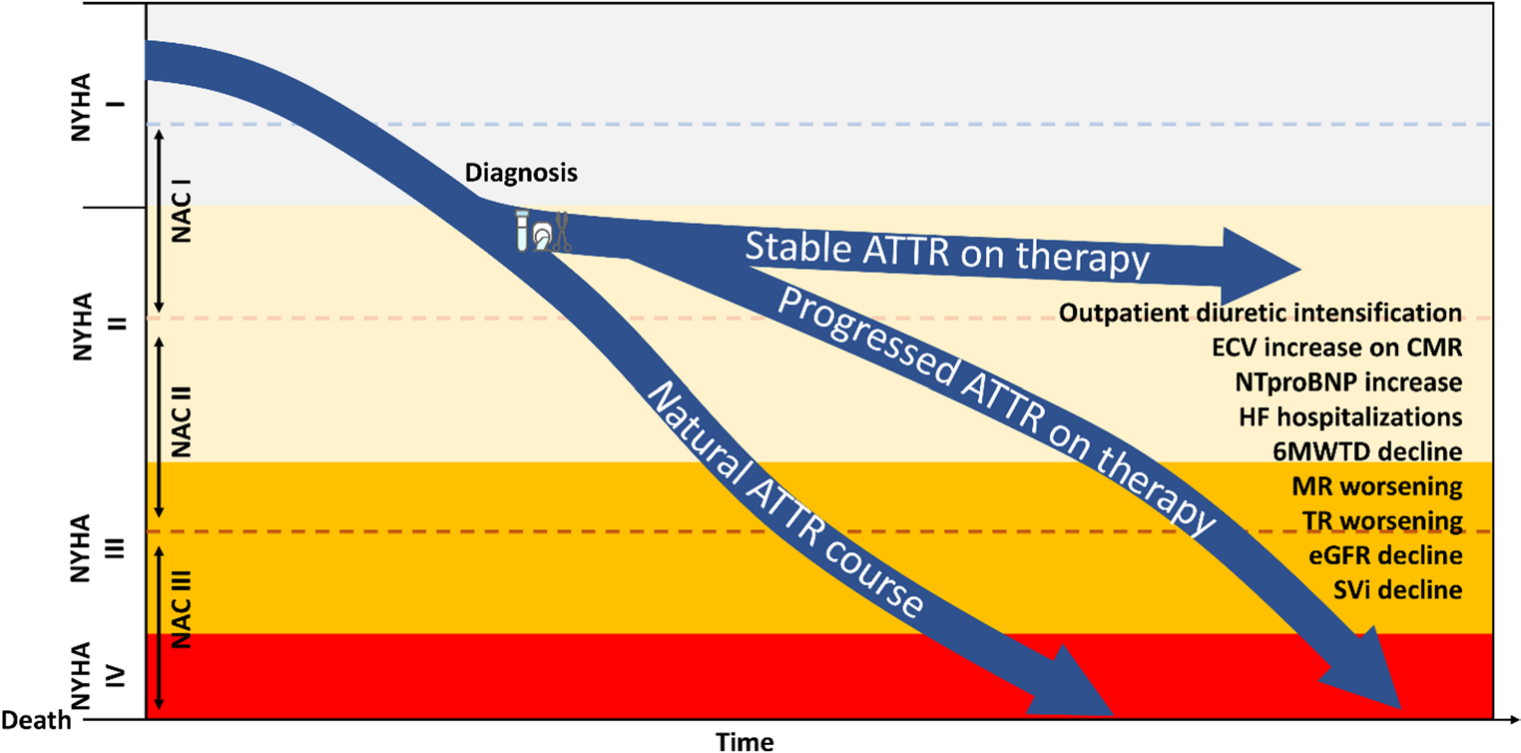
Disease progression detection in transthyretin amyloidosis with heart involvement. Abbreviations as in Figure 1 plus ATTR: transthyretin amyloidosis; CMR: cardiac magnetic resonance; ECV: extracellular volume; eGFR: estimated glomerular filtration rate; MR: mitral regurgitation; SVi: stroke volume indexed; TR: tricuspid regurgitation

In summary, to date, an easy, reliable and universal definition of ATTR disease progression is still lacking. Based on available evidence, markers reflecting haemodynamic congestion (ODI, NYHA class, NT-proBNP) and cardio-renal axis (eGFR) should be prioritized, even if also imaging-derived markers, particularly those reflecting amyloid load, may meaningfully contribute to risk stratification (Fig. 2). The increasing availability of disease-modifying therapies, in the absence of head-to-head comparative trials, presents both an opportunity and a clinical challenge in terms of treatment selection and therapeutic switching during follow-up. Early recognition of patients progressing despite treatment is critical for timely therapeutic adjustment, so that the development of a practical, reproducible, and universal framework for disease monitoring remains an urgent research priority.

## Data Availability

No datasets were generated or analysed during the current study.
